# YY1 Lactylation Aggravates Autoimmune Uveitis by Enhancing Microglial Functions via Inflammatory Genes

**DOI:** 10.1002/advs.202308031

**Published:** 2024-03-17

**Authors:** Jiaxing Huang, Xiaotang Wang, Na Li, Wei Fan, Xingran Li, Qian Zhou, Jiangyi Liu, Wanqian Li, Zhi Zhang, Xiaoyan Liu, Shuhao Zeng, Hui Yang, Meng Tian, Peizeng Yang, Shengping Hou

**Affiliations:** ^1^ Chongqing Key Laboratory of Ophthalmology, Chongqing Eye Institute; Chongqing Branch of National Clinical Research Center for Ocular Diseases The First Affiliated Hospital of Chongqing Medical University Chongqing 400016 China; ^2^ Department of Laboratory Medicine, Beijing Tongren Hospital Capital Medical University Beijing 100005 China; ^3^ Beijing Institute of Ophthalmology, Beijing Tongren Eye Center, Beijing Ophthalmology & Visual Sciences Key Laboratory, Beijing Tongren Hospital Capital Medical University Beijing 100730 China

**Keywords:** autoimmune uveitis, inflammation, lactylation, microglia, Yin‐Yang 1

## Abstract

Activated microglia in the retina are essential for the development of autoimmune uveitis. Yin‐Yang 1 (YY1) is an important transcription factor that participates in multiple inflammatory and immune‐mediated diseases. Here, an increased YY1 lactylation in retinal microglia within in the experimental autoimmune uveitis (EAU) group is observed. YY1 lactylation contributed to boosting microglial activation and promoting their proliferation and migration abilities. Inhibition of lactylation suppressed microglial activation and attenuated inflammation in EAU. Mechanistically, cleavage under targets & tagmentation （CUT&Tag） analysis revealed that YY1 lactylation promoted microglial activation by regulating the transcription of a set of inflammatory genes, including *STAT3, CCL5, IRF1, IDO1*, and *SEMA4D*. In addition, p300 is identified as the writer of YY1 lactylation. Inhibition of p300 decreased YY1 lactylation and suppressed microglial inflammation in vivo and in vitro. Collectively, the results showed that YY1 lactylation promoted microglial dysfunction in autoimmune uveitis by upregulating inflammatory cytokine secretion and boosting cell migration and proliferation. Therapeutic effects can be achieved by targeting the lactate/p300/YY1 lactylation/inflammatory genes axis.

## Graphical abstract

This study reveals that YY1 lactylation promotes microglial functions in autoimmune uveitis by upregulating inflammatory cytokine secretion and boosting cell migration and proliferation. Therefore, inhibiting YY1 lactylation genetically or pharmacologically alleviates symptoms of EAU and mitigates activation of microglia, indicating that therapeutic effects can be achieved by targeting the lactate/p300/YY1 lactylation and inflammatory gene (IRF1/IDO1/SEMA4D/CCL5/STAT3) axis.

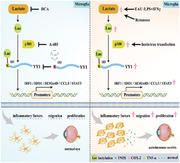

## 1 Introduction

Autoimmune uveitis (AU) is a prevalent immune disorder known for its potential to cause blindness. Patients often experience pronounced intraocular inflammation alongside a variety of systemic symptoms, including those associated with diseases such as Behcet's disease, Vogt‐Koyanagi‐Harada disease, and multiple sclerosis.^[^ ^1^ ^]^ The pathogenesis of AU involves the breakdown of the blood‐retinal barrier (BRB) and activation of Th17 cell and microglia.^[^ ^2^, ^3^, ^4^, ^5^ ^]^ Current treatments for AU typically include the administration of steroid hormones, novel immunosuppressants, and antimetabolic drugs. However, these treatments are suboptimal due to the complicated etiology and strong heterogeneity of AU.^[^ ^6^ ^]^ Consequently, patients with AU remain at risk of vision loss despite available therapeutic interventions.

Microglia are tissue‐specific macrophages primarily found in the central nervous system (CNS) and the retina.^[^ ^7^, ^8^, ^9^ ^]^ As resident immune cells, microglia play a crucial role in maintaining retinal homeostasis and guarding system development. Normally, they assume ramified shapes, facilitating the regulation of neuronal survival and interactions with other retinal cells. However, under pathological stimulation, microglia tend to transform from M0 to M1 phenotype, characterized by increased secretion of proinflammatory factors and enhanced abilities in migration and proliferation.^[^ ^8^, ^10^, ^11^ ^]^ This phenotype shift is considered a contributing factor in various retinopathies.^[^ ^12^, ^13^, ^14^ ^]^ For instance, in the initial stage of AU, activated microglia serve as key regulators, secreting proinflammatory cytokines such as inducible nitric oxide synthase （iNOS）, cyclooxygenase‐2 （COX‐2）, and tumor necrosis factor‐α （TNF‐α）, which lead to compromised integrity of BRB integrity and heightened recruitment of peripheral immune cells.^[^ ^3^, ^15^, ^16^ ^]^

Lactate, a by‐product of glycolysis, accumulates in situations demanding rapid adenosine triphosphate (ATP) generation or in response to oxygen deprivation.^[^ ^17^ ^]^ In many immune‐related diseases, lactate serves as a preferred energy source for maintaining immune cell functions, or acts as a signaling molecule to modulate their activity.^[^ ^18^ ^]^ Recently, a novel posttranscriptional modification known as lactylation has been identified, introducing a new field of gene expression regulation.^[^ ^19^, ^20^ ^]^ Lactylation has been reported to regulate macrophage polarization, modulate Treg cells generation and affect osteoblast differentiation.^[^ ^19^, ^21^, ^22^ ^]^ Ongoing research is exploring the role of lactylation in Alzheimer's Disease (AD), post‐myocardial infarction, and other immune‐related conditions.^[^ ^23^, ^24^ ^]^ However, whether lactylation influences microglial function and could therefore represent a potential a therapeutic target in AU remains unexplored.

The transcription factor YY1 is a DNA‐binding zinc finger protein that collaborates with chaperonins to regulate gene expression, either activating or repressing transcription.^[^ ^25^ ^]^ Research has revealed that YY1 was involved in inflammation‐related diseases, such as neuroinflammation, rheumatoid arthritis (RA), and nonalcoholic steatohepatitis.^[^ ^26^, ^27^, ^28^ ^]^ In a study on RA, blocking YY1 resulted in reduced IL‐6 production in LPS‐stimulated BV2 cells.^[^ ^29^ ^]^ Additionally, our previous study demonstrated that YY1 lactylation in microglia activated fibroblast growth factor 2 （FGF2） expression, consequently promoting pathological angiogenesis in retinopathy of prematurity (ROP).^[^ ^30^ ^]^ However, the connection between YY1 lactylation and AU remains unclear.

Based on these findings, we delved into the impact of YY1 lactylation on microglia in AU. We found that both the EAU and the microglia in LPS+IFN‐γ groups exhibited heightened levels of YY1 lactylation compared to the control groups, correlating with enhanced microglial activity. By elucidating the multidimensional alterations in YY1 lactylation, we further established its pivotal role in augmenting microglial functions. Mechanistically, we identified inflammatory genes, including *STAT3, CCL5, IRF1, IDO1*, and *SEMA4D*, as downstream targets of YY1. Our findings shed light on the functional significance of YY1 lactylation in microglia and its contribution to the exacerbation of AU, offering valuable insights into the modulation of inflammatory responses and the potential attenuation of AU progression. This study holds promise for informing future therapeutic strategies of AU.

## 2 Results

### 2.1 Elevated YY1 Lactylation was Associated with Microglia Activation in EAU

**Figure 1:**
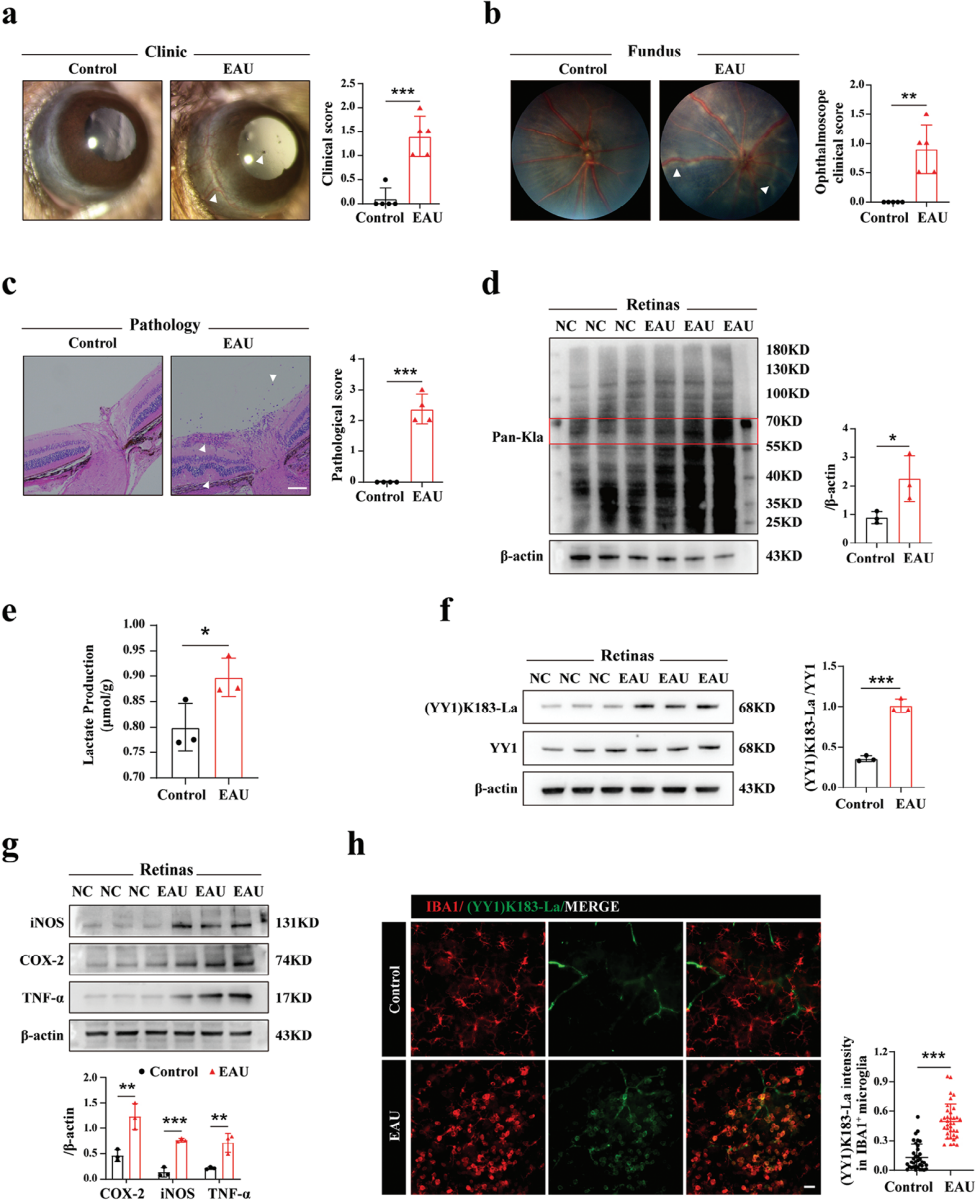
Elevated YY1 lactylation was associated with microglia activation in EAU. a) Representative images of slit lamp photography of control and EAU groups on day 14 and corresponding clinical scores (white arrows indicated conjunctival and ciliary congestion or anterior chamber inflammatory cells, n = 5). b) Representative fundus pictures of posterior pole of each group on day 14. Ophthalmoscope clinical scores were also exhibited (white arrows indicated focal lesions or curved blood vessels, n = 5). c) Representative H&E staining sections of retinas from each group on day 14 and corresponding pathological scores (white arrows indicated retinal tissue folds, vasculitis, or inflammatory cell infiltration; scale bar, 50 µm; n = 4). d) Quantification of retinal Pan‐Kla levels of the control and EAU groups on day 14 using WB (proteins in the range of 55–70 kD were marked; n = 3). e) Lactate production of retinal samples of each group (n = three independent experiments, at least six mice in each group). f,g) Quantification of retinal (YY1) K183‐La and YY1 protein levels and microglia M1 markers (iNOS, COX‐2, TNF‐α) measured using WB (n = 3). h) Representative images of (YY1) K183‐La co‐stained with microglia (IBA1) in retinas of EAU and control mice with quantification of (YY1) K183‐La intensity. Three fields per flat‐mount and three flat‐mounts per group were chosen for analysis (n = the number of IBA1^+^ cells; scale bar, 25 µm). Values were analyzed using the unpaired student's *t*‐test. *p < 0.05; **p < 0.01; ***p < 0.001.

Experimental autoimmune uveitis (EAU) was used as the classical model of AU.^[^ ^31^ ^]^ Female C57BL/6J mice (6‐8weeks of age) were subcutaneously injected with a mixture of interphotoreceptor retinoid‐binding protein （IRBP） and complete Freund's adjuvant （CFA）, then simultaneously received intraperitoneal pertussis toxin （PTX） injection (Sigma‐Aldrich, USA). On day 14, the EAU group exhibited more pronounced clinical and pathological scores. The anterior ocular segment images revealed that the EAU group showed specific conjunctival, ciliary congestion and infiltration of inflammatory cells (**Figure** **1**a). In fundus images, focal lesions and tortuous blood vessels were only observed in the EAU group (Figure 1b). Pathologically, the EAU group exhibited robust inflammatory pattern including retinal tissue foldings, vasculitis, and inflammatory cell infiltration, whereas the naive mice showed no pathological changes (Figure 1c).

Following the successful establishment of the EAU model, eyeballs were harvested for subsequent experiments on day 14. Notably, we observed significant increases in both lactate production and pan‐lysine lactylation （Pan‐Kla） protein levels in the retinas of the EAU group using a lactate content kit and western blotting (WB) (Figure 1d,e). Interestingly, the lactylation levels of proteins in the range of 55–70 kD showed a noticeable increase, suggesting a potential increase in YY1 (68 kD) lactylation (Figure 1d). To test this hypothesis, we employed a specific antibody (YY1) K183‐La to detect YY1 lactylation level. The EAU group showed elevated YY1 lactylation level while maintaining nearly unchanged YY1 expression compared to the control group (Figure 1f). Given the pivotal role of microglial activation in AU, we performed WB to assess protein expression of proinflammatory factors between two groups. We observed higher levels of iNOS, COX‐2, and TNF‐α in retinal tissues from the EAU group, indicating microglial activation in EAU (Figure 1g).

To ascertain whether other retinal cell components besides microglia may contribute to the higher protein levels of iNOS, COX‐2, and TNF‐α, we co‐stained the retinal flat‐mounts from the EAU group with DAPI, IBA1, and inflammatory factors (iNOS/COX‐2/TNF‐α). The results demonstrated a substantial overlap between IBA1‐positive cells and inflammatory‐factors‐positive cells, suggesting that the higher levels of inflammatory factors were primarily attributable to microglia (Figure S1h–j, Supporting Information). Considering the enhanced YY1 lactylation and the increased microglia activation observed via WB, we conducted a confocal immunofluorescence experiment to explore their correlation. Briefly, flat‐mounted retinas were double‐stained with antibodies against IBA1 and (YY1) K183‐La. As depicted in Figure 1h, IBA1‐positive cells in the EAU group showed an increase in cell numbers and manifested an activation morphology (amoeboid), whereas those in the control group presented resting shapes (ramified). Collectively, the in vivo results suggested an association between YY1 lactylation and microglial activation in EAU.

### 2.2 YY1 Lactylation Positively Correlated with Microglia Activation, Migration, and Proliferation In Vitro

**Figure 2:**
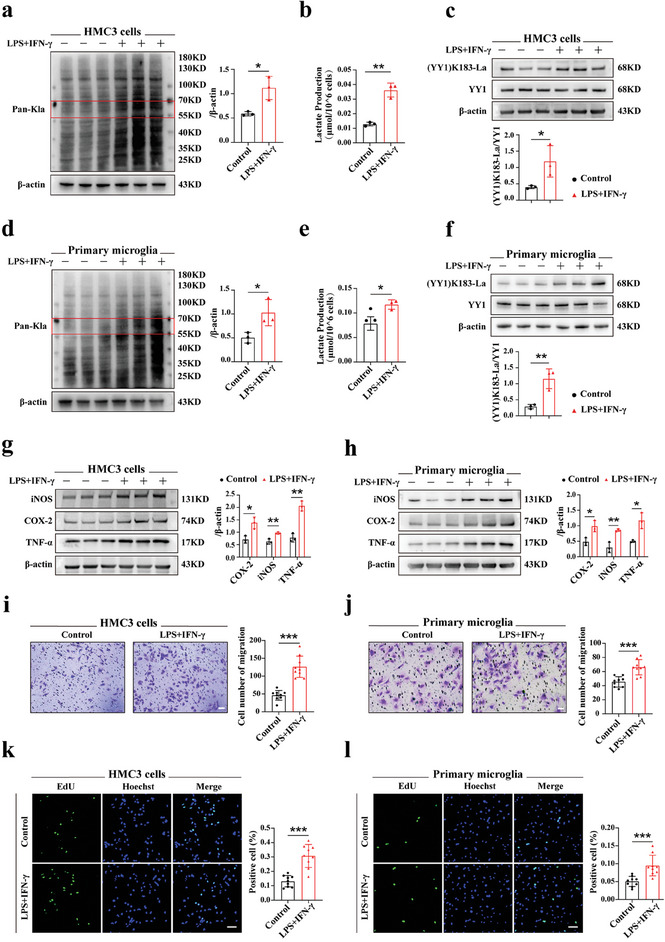
YY1 lactylation positively correlated with microglia activation, migration, and proliferation in vitro. a) Quantification of Pan‐Kla levels in control and LPS+IFN‐γ groups using HMC3 cells (n = 3). b) Lactate production of HMC3 cells in two groups (n = three independent experiments, at least 1×10^7^ cells per group). c) Quantification of (YY1) K183‐La and YY1 protein levels between two groups using HMC3 cells (n = 3). d‐f) Using primary microglia, Pan‐Kla level, lactate production, and protein levels of (YY1) K183‐La and YY1 were measured and analyzed in the same way as HMC3 cells. g,h) Microglia inflammatory factors (iNOS, COX‐2, TNF‐α) were measured using WB in HMC3 cells and primary microglia respectively (n = 3). i,j) Using two cell lines, the migration of microglia was evaluated using the Transwell assays (n = three independent experiments, three images for each group; scale bar, 25 µm). k,l) Proliferation of microglia was measured using the EdU assay in both cell lines (n = three independent experiments, three images for each group; scale bar, 50 µm). Unpaired student's *t*‐test was adopted for analyses. *p < 0.05; **p < 0.01; ***p < 0.001.

To further elucidate the relationship between microglial function and YY1 lactylation, in vitro experiments were conducted due to the diverse cell populations present in the retina. Human microglia clone 3 (HMC3) cells and primary microglia were evenly cultured in 6‐well plates, and randomly assigned to two groups. The control groups were cultured in serum‐free EMEM culture medium, while the experimental groups were additionally treated with lipopolysaccharide （LPS） (1 µg mL^−1^) and interferon‐γ （IFN‐γ） (500 ng mL^−1^) for 24 h. Consequently, both cell lines showed higher Pan‐Kla protein expression and enhanced lactate production in the experimental groups, suggesting an elevation in total lactylation level due to the higher lactate content (**Figure** **2**a,b,d,e). Additionally, we observed that LPS+IFN‐γ stimulation significantly increased lactylation of YY1 and proteins in the range of 55–70 kD in both cell lines (Figure 2c,f).

To investigate how microglial functions changed in response to up‐regulated YY1 lactylation, we examined the protein levels of inflammatory cytokines, including iNOS, COX‐2 and TNF‐α, using Western blotting (WB) analysis. The results indicated a marked increase in these cytokines after stimulation, indicating a microglial activation (Figure 2g,h). Moreover, we observed increased number of migrated cells in the experimental group, indicating that LPS+IFN‐γ strengthened microglial migration ability (Figure 2i,j). Additionally, microglia that received LPS+IFN‐γ showed reinforced proliferation ability as demonstrated by the EdU assay (Figure 2k,l). These findings revealed that a positive correlation between YY1 lactylation and microglial activation, migration, and proliferation.

### 2.3 YY1 Lactylation Altered by Lactate Generation Affects EAU

Given that lactylation is mediated by lactate content, we employed Rotenone (mitochondrial complex I inhibitor) or sodium dichloroacetate （DCA） (pyruvate dehydrogenase kinase inhibitor) to increase or decrease lactate generation respectively.^[^ ^32^ ^]^ EAU mice were randomly divided into three groups, with each group receiving intraperitoneal administration of a moderate amount of DMSO (0.1%), Rotenone (1.5 mg kg^−1^) or DCA (200 mg kg^−1^) on day 9 for five consecutive days. On day 14, mice were sacrificed after obtaining images of anterior segment and fundus. Eyeballs were utilized for hematoxylin and eosin (H&E) staining, immunofluorescence staining, and protein extraction.

**Figure 3:**
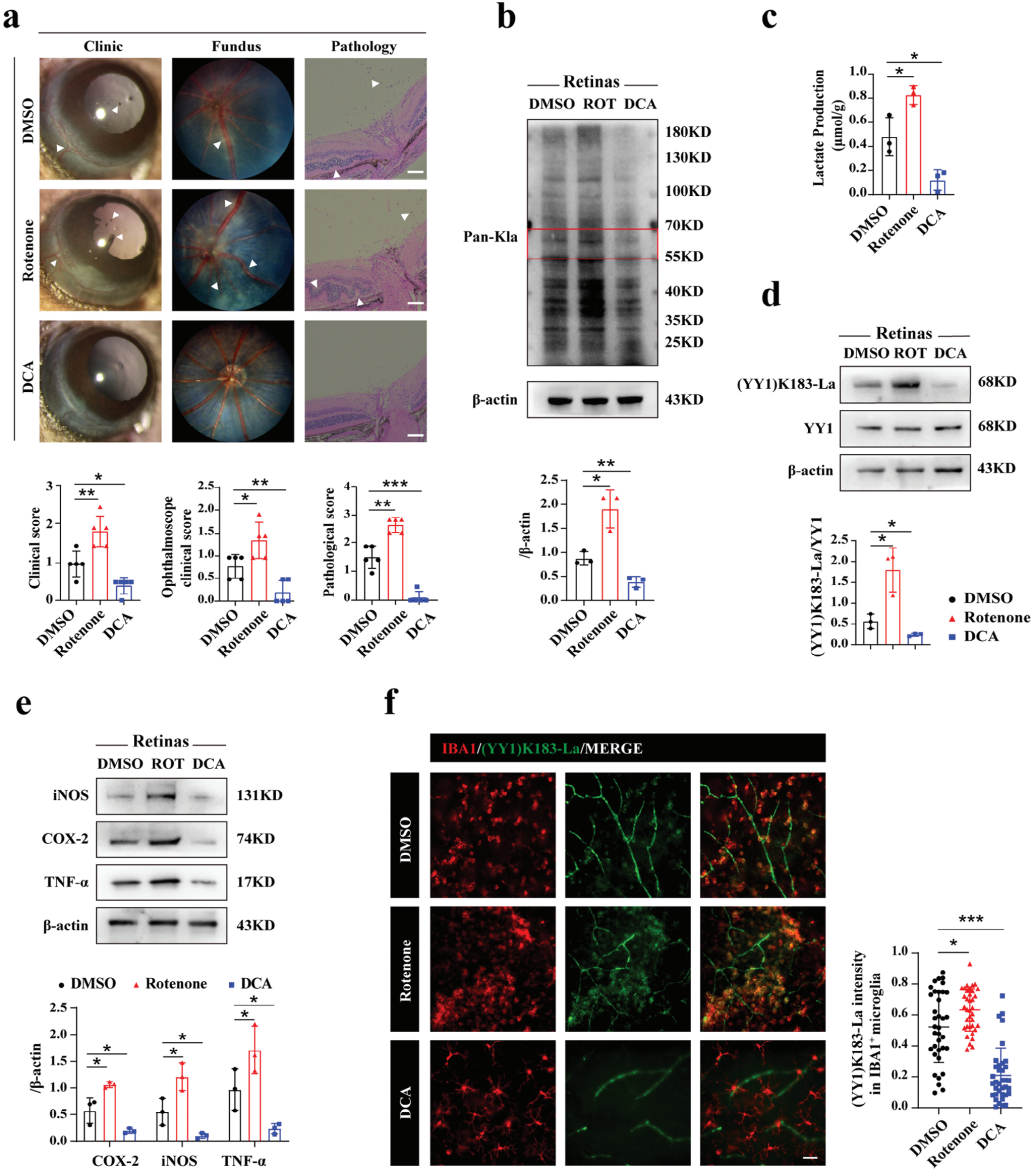
YY1 lactylation altered by lactate generation affects EAU. a) Representative images of slit lamp photography, fundus pictures, and H&E staining sections in three groups (DMSO/Rotenone/DCA) with corresponding statistics (in clinic images, white arrows indicated infiltration of inflammatory cells, conjunctival and ciliary congestion or peripheral anterior synechia; in fundus images, white arrows indicated lesion regions and curved vessels; in pathological images, white arrows indicated inflammatory cells, retinal folds or retinal detachment; scale bar, 50 µm). b) Quantification of retinal Pan‐Kla level in DMSO/Rotenone/DCA groups (n = three independent experiments). c) Lactate production of each group using retinas (n = three independent experiments, at least six mice per group). d) Representative images of western blotting results for (YY1) K183‐La and YY1 in whole retinal extracts and corresponding statistics (n = 3). e) Quantification of microglia M1 markers (iNOS, COX‐2, TNF‐α) in each group using retinal extracts (n = 3). f) Representative images of (YY1) K183‐La co‐stained with microglia (IBA1) in retinas of each group with quantification of (YY1) K183‐La intensity. (n = the number of IBA1^+^ cells; scale bar, 25 µm). One‐way ANOVA; *p < 0.05; **p < 0.01; ***p < 0.001.

The Rotenone group exhibited more severe clinical symptoms and pathological manifestations, including peripheral anterior synechia, enhanced infiltration of inflammatory cells, enlarged lesion regions, increased retinal folds, and retinal detachment (**Figure** **3**a). In contrast, DCA appeared to alleviate EAU both clinically and pathologically. As expected, Pan‐Kla level, lactate production and YY1 lactylation were influenced by Rotenone and DCA (Figure 3b–d). Notably, Rotenone significantly enhanced the protein expression of inflammatory cytokines, including iNOS, COX‐2 and TNF‐α, whereas DCA ameliorated this effect (Figure 3e). Furthermore, based on the results of double‐label immunofluorescence, we found that the Rotenone group displayed more activated microglia and enhanced YY1 lactylation, whereas microglia in the DCA group reverted into resting state with no obvious colocalization detected (Figure 3f). In summary, YY1 lactylation, mediated by lactate generation, positively controls microglial activation, hereby influencing the progression of EAU.

### 2.4 YY1 Lactylation Altered by Lactate Generation Changed Microglia Functions In Vitro

**Figure 4:**
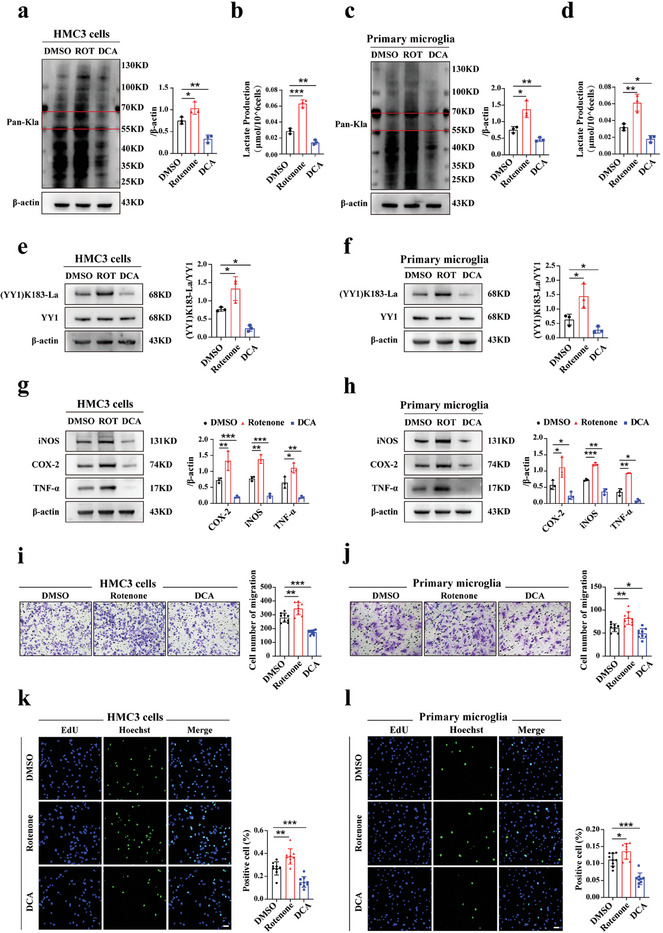
YY1 lactylation altered by lactate generation changed microglia functions in vitro. a) Quantification of Pan‐Kla level in DMSO/Rotenone/DCA groups after 24 h LPS+IFN‐γ stimulation using HMC3 cells (n = 3). b) Lactate production of HMC3 cells in three groups after LPS+IFN‐γ treatment for 24 hours (n = three independent experiments, at least 1×10^7^ cells per group). c,d) Using primary microglia, Pan‐Kla level and lactate production of each group were assessed in the same way as HMC3 cells (n = 3). e–h) In both cell lines, inflammatory factors (iNOS, COX‐2, TNF‐α), YY1, and (YY1) K183‐La protein expression levels were measured using WB after 24 hours LPS+IFN‐γ stimulation with corresponding statistics (n = 3). i,j) Using two cell lines, migration abilities of microglia in each group were evaluated using the Transwell assays (n = three independent experiments, three images for each group; scale bar, 25 µm). k,l) Proliferation of HMC3 cells or primary microglia was respectively measured by EdU assays in three groups (n = three independent experiments, three images for each group; scale bar, 50 µm). One‐way ANOVA; *p < 0.05; **p < 0.01; ***p < 0.001.

In vitro, HMC3 cells/primary microglia were randomly assigned to three groups. Each group was stimulated with LPS (1 µg mL^−1^) and IFN‐γ (500 ng mL^−1^) and received a moderate amount of DMSO, Rotenone, and DCA respectively. After 24 h, the microglia were subjected to subsequent tests. Consistent with the in vivo results, Rotenone and DCA mediated Pan‐Kla levels, lactate production, and YY1 lactylation in both HMC3 cells and primary microglia (**Figure** **4**a–f).

To further detect changes in microglial function, we performed WB, Transwell, and EdU assays. It appeared that Rotenone promoted the expression of inflammatory factors including iNOS, COX‐2 and TNF‐α while DCA decreased those (Figure 4g,h), thereby reinforcing the positive correlation between YY1 lactylation and microglial activation. The migration and proliferation abilities of microglia were promoted after Rotenone treatment and were abated by DCA administration (Figure 4k,l). Generally, these findings underscore the role of YY1 lactylation in regulating microglial function in vitro.

### 2.5 YY1 de‐Lactylation Alleviated Microglial Functions

**Figure 5:**
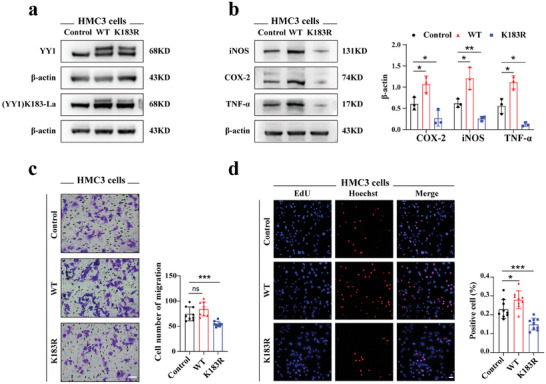
YY1 de‐lactylation alleviated microglial functions. a) Representative images of WB results for YY1 and (YY1) K183‐La in control, WT, and K183R groups using HMC3 cells stimulated with LPS+IFN‐γ for 24 hours. The upper band represented the FLAG‐tagged YY1 while the lower band showed the untagged YY1. b) Quantification of inflammatory cytokine (iNOS, COX‐2, TNF‐α) levels in control, WT, and K183R groups using HMC3 cells stimulated with LPS+IFN‐γ for 24 hours. c) Representative images of the Transwell assay results showing migration abilities of control, WT, and K183R groups (n = 3; scale bar, 25 µm). d) Proliferation of microglia was measured using EdU assays in three groups (n = three independent experiments, three images for each group; scale bar, 50 µm). One‐way ANOVA; *p < 0.05; **p < 0.01; ***p < 0.001.

Adjusting lactate generation by Rotenone and DCA undoubtedly regulates YY1 lactylation. To explore this further, we sought to genetically control YY1 lactylation. By mutating lysine (K) to arginine (R), the de‐lactylation state of YY1 can be achieved. HMC3 cells were transfected with lentivirus carrying cDNA of FLAG‐tagged YY1 WT or YY1 K183R mutants. We observed notably higher YY1 protein expression in the WT and K183R groups and reduced YY1 lactylation level in the K183R group (**Figure** **5**a). Next, each group received LPS+IFN‐γ for 24 hours. WB results revealed that the WT group showed improved YY1 lactylation and inflammatory cytokine expression, whereas the K183R group showed a sharp decline, which confirmed that YY1 lactylation positively regulated microglial activation (Figure 5b). Regarding other microglial functions, Transwell assays showed that the K183R group strongly alleviated microglial migration, while no obvious differences were observed in the WT group (Figure 5c). EdU assays showed that microglial proliferation was regulated by YY1 lactylation (Figure 5d). In conclusion, these results indicated that YY1 lactylation plays a crucial role in microglial activation, migration, and proliferation.

### 2.6 p300 Affects Microglia Functions In Vitro

**Figure 6:**
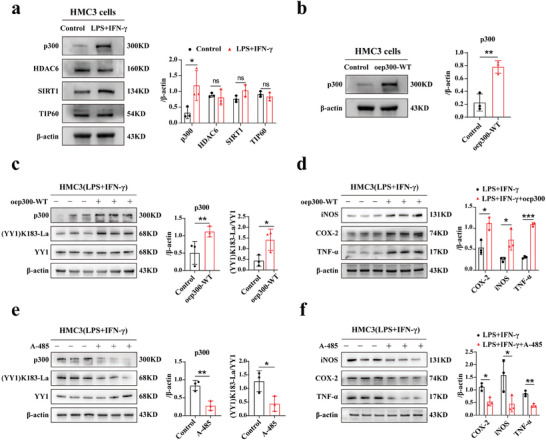
Effect of p300 on microglial function in vitro. a) Representative images of WB results for lacytlation writers/erasers (p300/HDAC6/SIRT1/TIP60) in the control and LPS+IFN‐γ groups with corresponding statistics (n = 3). b) p300 protein expression was measured using WB in control and oep300‐WT groups (n = 3). c,d) The protein expression of p300, YY1, (YY1) K183‐La, and inflammatory factors (iNOS, COX‐2, TNF‐α) in HMC3 cells and oep300‐WT cells both exposed to LPS+IFN‐γ for 24 h (n = 3). e,f) The protein expression of p300, YY1, (YY1) K183‐La, and inflammatory factors (iNOS, COX‐2, TNF‐α) in HMC3+LPS+IFN‐γ and HMC3+LPS+IFN‐γ+A‐485 groups (n = 3). Unpaired student's *t‐*tests were used for the analysis. *p < 0.05; **p < 0.01; ***p < 0.001.

To date, specific regulators responsible for lactylation have not been fully explored. We hypothesized that lactylation might share regulators with lysine acylation. To explore this, we examined the protein levels of acknowledged acylation modification writers (Tip60 and p300) and erasers (HDAC6 and SIRT1) using WB.^[^ ^33^, ^34^ ^]^ The results showed that p300 was strongly upregulated after LPS+IFN‐γ stimulation, while no significant variances were found in other groups (**Figure** **6**a).

P300 was selected as a potential regulator of YY1 lactylation and co‐immunoprecipitation (Co‐IP) results showing that p300 bound to YY1 confirmed the interaction between both proteins in microglia (Figure S2b, Supporting Information). To further explore the role of p300 in YY1 lactylation, HMC3 cells were treated with a p300 inhibitor (A‐485) and a p300 overexpression lentivirus to establish p300 underexpression and overexpression models, respectively. As shown in Figure 6b, the presence of the oep300‐WT model was confirmed using WB. YY1 lactylation levels were in accordance with p300 overexpression, and inflammatory factors were also increased (Figure 6c,d). Conversely, p300, which is under‐expressed by A‐485, downregulated YY1 lactylation and alleviated microglial activation. Taken together, these results indicated that p300 can regulate microglial activation. Furthermore, we observed an obvious increase in p300 protein level in the EAU group using retinal tissues (Figure S1b, Supporting Information). After vitreously injecting the EAU mice with A‐485, the symptoms of EAU were mitigated to some extent. There were no anterior synechia or inflammatory cell infiltration found in the anterior ocular segment images, lower pathological scores, and less secretion of inflammatory factors following A‐485 treatment (Figure S1c–e, Supporting Information).

### 2.7 Several Inflammatory Genes are Downstream Targets of YY1 Lactylation

**Figure 7:**
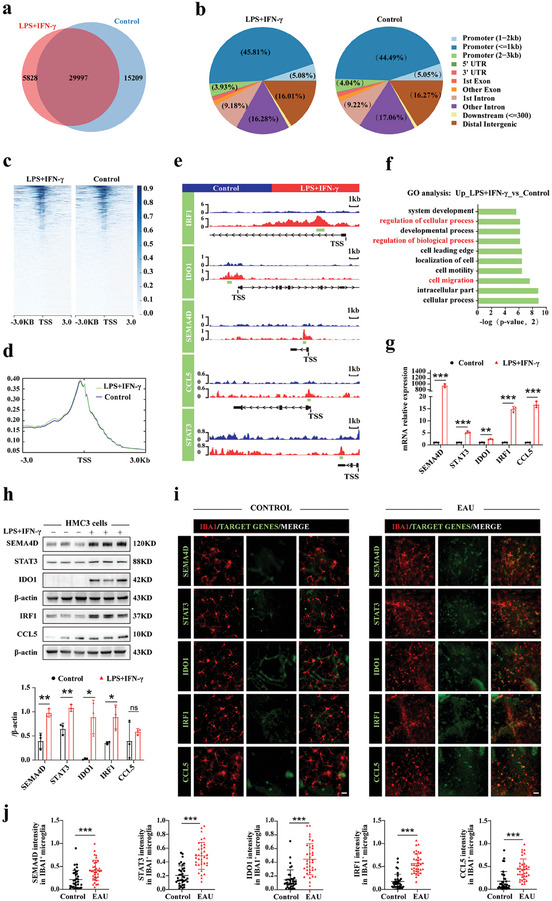
Several inflammatory genes were downstream targets of YY1 lactylation. a) Venn diagram showing the overlap peaks between control and LPS+IFN‐γ groups in HMC3 cells. b) Genome wide distribution of YY1 binding peaks in control and LPS+IFN‐γ groups, respectively. c) The binding density of YY1 was visualized using deepTools. The heatmap presented the CUT&Tag counts on different peaks in HMC3 cells between control and LPS+IFN‐γ groups, ordered by signal strength. d) Metagene analyses of YY1 coverages at TSS. Regions selected from TSS (± 3 kb). e) Genome browser tracks of CUT&Tag signal at the representative target gene loci (track length:17 kb). The green rectangles indicated the up‐peak regions on target‐genes promotors (≤ ± 3 kb to TSS). f) GO analysis showed that upregulated genes primarily participate in bioprocesses, as exhibited in the histogram. g) Quantification of mRNA expression of identified genes (*SEMA4D/STAT3/IDO1/IRF1/CCL5*) in control and LPS+IFN‐γ groups, measured by RT‐qPCR. h) Selected‐gene protein expressions measured using WB between two groups with corresponding statistics (n = 3). i,j) Representative images of selected‐gene co‐stained with microglia (IBA1) in retina of EAU and control mice with quantification of *SEMA4D/STAT3/IDO1/IRF1/CCL5* intensity. (n = the number of IBA1^+^ cells; scale bar, 25 µm). Values were analyzed using the unpaired student's *t*‐test. *p < 0.05; **p < 0.01; ***p < 0.001.

YY1, a transcription factor that regulates gene expression,^[^ ^25^ ^]^ undergoes epigenetic modifications implicated in gene mediating.^[^ ^30^, ^35^, ^36^ ^]^ In a study on chronic hepatitis, YY1 degradation via ubiquitination activated the HNF4 α/MiR‐122/CCL2 pathway,^[^ ^35^ ^]^ while YY1 phosphorylation at S118 induced atherosclerosis.^[^ ^36^ ^]^ Our previous study showed that YY1 lactylation contributed to elevated FGF2 expression.^[^ ^30^ ^]^ Building on these findings, CUT&Tag analysis was performed to identify the downstream target genes of YY1 lactylation. Briefly, HMC3 cells were divided into two groups: the control group was cultured in serum‐free EMEM and the experimental group was stimulated with LPS+IFN‐γ additionally for 24 h. Using CUT&Tag antibodies against YY1, we identified 29997 overlapped peaks between the two groups (**Figure** **7**a). Most peaks were located within 3 kb of the TSS (promoter region) (Figure 7b–d).

Based on the evident microglial functional changes in our experiments and the recognized role of inflammation in AU, we systematically examined up‐regulated genes in fold‐change order with previous studies to weigh their potential in managing microglial functional changes in AU. Five inflammatory genes were selected: *IRF1* (5.68 fold‐change), *IDO1* (4.08 fold‐change), *SEMA4D* (4.60 fold‐change), *CCL5* (2.25 fold‐change), *STAT3* (2.07 fold‐change). As illustrated in Figure 7e, Integrative Genomics Viewer (IGV) images showed that the promotor regions (≤±3 kb to TSS) of *IRF1/IDO1/SEMA4D/CCL5/STAT3* were considerably affected by YY1, suggesting that YY1 lactylation may enhance transcription of these genes. According to previous studies, *IRF1* positively regulates microglial activation, amplifies retinal inflammation, and enhances the migratory abilities of multiple cancer cells.^[^ ^37^, ^38^, ^39^ ^]^ *IDO1* was closely related to the enhanced migration and proliferation of cancer cells and participates in several immune and inflammatory diseases.^[^ ^40^, ^41^, ^42^, ^43^ ^]^ *SEMA4D* reportedly activated microglia in neurodegenerative and infectious diseases.^[^ ^44^, ^45^ ^]^ Moreover, *SEMA4D* has been shown to promote the migration of HUVECs, eosinophils and the proliferation of cancer cells.^[^ ^46^, ^47^, ^48^ ^]^ *CCL5*, characterized as an M1‐type marker of microglia, exerts a significant influence on microglial polarization,^[^ ^49^, ^50^ ^]^ with research indicating its ability to enhance migration and proliferation of cancer cells.^[^ ^51^, ^52^ ^]^ *STAT3*, a key regulator of the JAK/STAT3 pathway, is involved in the progression of several inflammatory diseases.^[^ ^53^, ^54^ ^]^ In AU, *STAT3* promotes the expansion of Th17 cells and exacerbates uveitis,^[^ ^55^ ^]^ while also mediating cell migration and proliferation.^[^ ^56^, ^57^ ^]^ As these target genes can regulate several biological functions, the changes observed in microglial functions were closely connected and influenced each other. These selected genes were involved in several top pathways according to GO analysis, including cell migration, regulation of cellular processes, and regulation of biological processes (Figure 7f).

To validate the involvement of candidate genes in EAU, we stimulated HMC3 cells with LPS+IFN‐γ for 24 hours and collected proteins and RNAs. RT‐qPCR results revealed a sharp increase in the RNA expression of the selected genes, and WB demonstrated significant elevations in *STAT3*, *IDO1, IRF1* and *SEMA4D* as well. However, the protein expression of *CCL5* showed only a slight enhancement (Figure 7g,h). To detect the target gene expression in vivo, we double‐stained retinas with IBA1 and target gene antibodies to measure alterations between the control and EAU groups (Figure 7i). All genes were greatly promoted in accordance with activated microglia in the EAU group, while the co‐localization and immunofluorescence intensity of the target genes were much weaker in the control group (Figure 7j). To determine whether these genes were mainly regulated by YY1, we collected protein samples from HMC3 microglia which were transfected with lentivirus carrying cDNA of FLAG‐tagged YY1 WT or YY1 K183R mutants and stimulated with LPS+IFN‐γ for 24 h. WB results indicated that after YY1 mutation the downstream targets protein expressions were decreased accordingly (Figure S1a, Supporting Information). Furthermore, we confirmed that these genes were mediated by YY1 lactylation level through lactate generation interventions (DMSO/Rotenone/DCA) (Figure S1f, Supporting Information). In conclusion, these results indicated that YY1 lactylation influences microglial functions through the regulation of *IRF1, IDO1, SEMA4D, CCL5 and STAT3*.

## 3 Discussion

**Figure 8:**
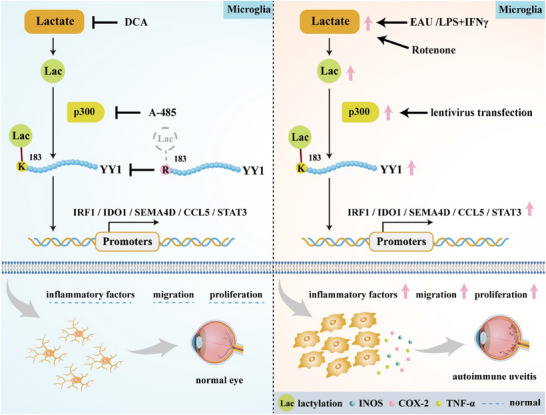
The schematic diagram illustrated the molecular mechanism of how YY1 lactylation in microglia contributed to inflammation in AU.

Our studies demonstrated that microglial functions were promoted in AU through YY1‐lactylation‐mediated upregulation of several inflammatory genes. The results indicated that YY1 lactylation showed a positive correlation with microglial function, contributing to microglial activation, and enhancing microglial mobility and proliferation. Additionally, the results suggested that p300 positively regulated YY1 lactylation and promoted changes in microglial function. Moreover, through CUT&Tag analyses, five inflammatory genes were selected as target genes mediated by YY1 lactylation. Taken together, we discovered a novel mechanism that regulates microglial function in AU. Through intervention of the lactate/p300/YY1 lactylation/inflammatory gene (*IRF1/IDO1/SEMA4D/CCL5/STAT3*) axis, it may be possible to reverse proinflammatory microglial features and potentially alleviate AU progression.(**Figure** **8**).

Accumulating evidence suggests that microglia can be activated under pathological conditions, leading to the release of proinflammatory mediators such as iNOS, COX‐2, TNF‐α, etc.^[^ ^58^ ^]^ In AU, activated microglia have been implicated as key instigators of early‐stage retinal inflammation,^[^ ^3^, ^8^ ^]^ acting as “culprits” by promoting breakdown of the BRB, secreting proinflammatory cytokines, and recruiting peripheral immune cells.^[^ ^8^ ^]^ Activated microglia also created an inflammatory cascade since these cytokines were capable of reactivating microglia.^[^ ^59^, ^60^ ^]^ Crucial as microglial activation was, the underlying mechanism remains elusive. YY1, a transcription factor that regulates gene expression, has been studied extensively in many types of cancer and angiogenesis.^[^ ^23^, ^61^ ^]^ However, its role in autoimmune diseases remains largely unexplored.^[^ ^62^ ^]^ Among the preliminary screening of target protein using lactylated modified pan‐antibodies, YY1 caught our attention since its lactylation level was largely upregulated and by its novelty in AU and complexity in gene expression regulation.^[^ ^25^, ^63^, ^64^ ^]^ The results also detected elevation of lactylation in other proteins, further studies are needed to investigate on other possible lactylated genes besides YY1. Using the specialized YY1‐K183la antibody (YY1‐K183La), our in vivo and in vitro results suggested that YY1 lactylation and microglial activation co‐existed in AU. Through the regulation of YY1 lactylation by interventions involving lactate content, YY1 mutation and p300 regulation, we observed that microglial activation was remarkably alleviated by YY1 lactylation downregulation. Additionally, CUT&Tag analysis revealed that YY1 lactylation upregulates several inflammatory genes closely related to changes in microglial functions including migration, proliferation and activation. These functional changes were closely connected and influenced each other. It is reported that activated microglia enhanced proliferation and migration abilities to exacerbate inflammation, while the inflammatory cascade caused a positive feedback loop on microglial activation.^[^ ^60^, ^65^ ^]^ In conclusion, these results indicated that YY1 lactylation was responsible for microglial activation in AU.

Since Zhang et al ^[^ ^19^ ^]^ discovered that histone lactylation affects macrophage polarization, numerous studies have explored the potential implications of lactylation. It has been investigated in multiple cancers, inflammatory diseases and immune illnesses.^[^ ^22^, ^24^, ^30^ ^]^ To date, the relationship of AU and lactylation remains unclear. Our study found that YY1 lactylation played a crucial role in promoting pro‐inflammatory microglial functions,^[^ ^66^ ^]^ therefore, it was regarded as a potential pathogenic target for AU. Effective regulation of lactylation was essential in our experiments. The intervention strategies were listed as follows: First, given that lactate, a product of glycolysis, acts as a precursor that stimulates lactylation, and drawing from existing literature, we used Rotenone (mitochondrial complex I inhibitor) and DCA (pyruvate dehydrogenase kinase inhibitor) to induce the upregulation and downregulation of lactylation, respectively.^[^ ^32^ ^]^ Second, for accuracy, a mutation in the target lysine site at the genetic level was adopted. Since our previous study detected a single lysine lactylation site (K183) in YY1, the lysine at site 183 was mutated to arginine to attain a de‐lactylation state of YY1.^[^ ^30^ ^]^ Third, given that the specific writers or erasers exclusively mediating lysine lactylation have yet to be identified, we selected known writers (Tip60 and p300) and erasers (HDAC6 and SIRT1) as candidate regulators.^[^ ^33^, ^34^ ^]^ P300 was chosen as most evidently altered after stimulation with LPS+IFN‐γ. Similarly, the lysine acetylase p300 has been reported to catalyze the transfer of the lactyl group from lactyl‐CoA to histones in a cell‐free system, and p300 catalyzes the lactylation of HMGB1 and Snail1.^[^ ^67^, ^68^ ^]^ Subsequently, p300 lentivirus and the inhibitor A‐485 were used as enhancers and suppressors of YY1 lactylation, respectively. By combining these methods, we achieved multidimensional alteration of YY1 lactylation.

YY1 is also involved in several inflammatory diseases. A previous study indicated that YY1 promoted NF‐κB‐mediated overexpression of inflammatory cytokines in rheumatoid arthritis.^[^ ^69^ ^]^ Moreover, YY1 affected Th17 cell activation and positively regulated the STAT3 pathway.^[^ ^70^ ^]^ Concurrently, emerging research on epigenetic modifications of YY1 includes YY1 phosphorylation at S118, which contributes to atherosclerosis, and YY1 deacetylation, implicated in renal fibrosis.^[^ ^36^, ^71^ ^]^ Despite AU being a prototypical inflammatory condition necessitating novel treatments, no studies have linked AU with YY1 lactylation. For the first time, we discovered that YY1 lactylation is a potential pathogenic factor of AU and identified five downstream target genes (*IRF1/IDO1/SEMA4D/CCL5/STAT3*), that shed light on the disease etiology. As we found increased protein levels in a series of inflammatory factors (iNOS, COX‐2, TNF‐α, MCP1, IL‐1β and IL‐6), we wondered whether YY1 regulated these genes (Figure 2g; Figure S2a, Supporting Information). However, CUT&Tag results showed that these inflammatory genes didn't directly bind to YY1. The IGV images illustrated that there was no obvious elevation in these genes between the control and the LPS+IFN‐γ groups (Figure S2c, Supporting Information). It's more possible that the elevation of these inflammatory proteins resulted from a complex inflammation network than directly induced by YY1. STAT3 was a downstream target gene of YY1 and reported to be involved AU.^[^ ^72^, ^73^ ^]^ Interestingly, many inflammatory factors can be affected by STAT3 pathway, so it's possible that STAT3 upregulation caused by YY1 further affected the levels of iNOS, COX‐2, TNF‐α, MCP1, IL‐1β and IL‐6.^[^ ^74^, ^75^ ^]^ It is reported that mouse YY1 was involved in macrophage polarization, pathological angiogenesis, tumorigenesis, and the modulation of immune processes.^[^ ^61^, ^76^, ^77^ ^]^ Similarly, human YY1 has been reported to participate in cancer development, modulation of neuroinflammation, and Th17 cell differentiation.^[^ ^61^, ^78^, ^79^ ^]^ To further explore the translatability between human and mouse YY1, we compared both YY1 protein sequences on the UniProt website. The result showed that they shared high degree of homology (>98%), suggesting that our findings in mouse YY1 were largely representative of human YY1.

To validate our results in AU patients, we included three patients in the active phase of AU as well as three healthy controls, and their serums were collected for lactate content measurement by the LA Content Assay Kit (Solarbio, BC2235). The results showed that serum lactate levels were significantly raised in patients with AU, which suggested that lactate may play a critical role in the development of uveitis (Figure S1g, Supporting Information). In conclusion, the pro‐inflammatory role of YY1 lactylation in AU that we discovered may contribute to developing target treatments for AU.

Despite the significant findings, our study has several limitations. First, although the YY1‐K183 mutation greatly influenced microglial function in HMC3 cells and primary microglia, whether the same mutation was capable of slowing AU progression in vivo and in uveitis patients and human primary microglia required further investigation. To better elucidate this effect, studies investigating knock‐in or overexpression of WT/delactylated YY1 forms in retinal microglia are necessary. Second, we selected five downstream target genes regulated by YY1 lactylation, which are reportedly inflammation‐related, with some known to mediate migration and proliferation. Simply assuming that they uniformly contribute to exacerbating AU symptoms may obscure our understanding when selecting specific treatments. Therefore, further research to determine the dominant gene among these targets would be beneficial.

## 4 Experimental Section

### Patients

This study was performed in accordance with the tenets of the Declaration of Helsinki, and was approved by the Ethics Committee of the First Affiliated Hospital of Chongqing Medical University (Approval number:2021‐222). Patients in the active phase of AU and healthy controls were included. All participants signed an informed consent form at the start of the study. Serum samples were used for lactate content analyses.

### Reagents

Rotenone and Sodium Dichloroacetate (DCA) were purchased from Aladdin (Shanghai, China). A‐485 was obtained from MCE (New Jersey, USA). Human IRBP_651‐670_ (LAQGAYRTAVDLESLASQLT) (purity>98%) was synthesized by Sangon Biotech (Shanghai, China). Pertussis toxin (PTX) was obtained from Sigma–Aldrich (St. Louis, Missouri, USA). A heat‐killed *Mycobacterium tuberculosis* strain H37Ra was purchased from BD Biosciences (Franklin Lake, NJ, USA). Lipopolysaccharide, DMSO, and complete Freund's adjuvant (CFA) were purchased from Sigma–Aldrich. human IFN‐γ was obtained from PeproTech (Cranbury, NJ, USA).

### Animals

Mice were obtained from the Experimental Animal Center of Chongqing Medical University (female, C57BL/6J, 6–8 weeks old) and bred in a specific pathogen‐free environment. All experiments strictly abided by the Ethics Committee of the First Affiliated Hospital of Chongqing Medical University and conformed to the ARVO Statement for the Use of Animals in Ophthalmic and Vision Research (2019‐296).

### EAU Model and DMSO/DCA/Rotenone Treatment

Human IRBP_651–670_ (500 mg) was dissolved in PBS (1 ml) and *M. tuberculosis* strain H37Ra (40 mg) was dissolved in Freund's adjuvant (1 ml). Then, IRBP and Freund's adjuvant were emulsified with an equal volume for an hour. Mice received subcutaneous injections of the above‐mentioned mixtures at six sites and intraperitoneal injections of PTX (1 µg) to induce the EAU model. After immunization for nine days, the mice were randomly distributed into three groups and subjected to intraperitoneal injection with DCA (200 mg kg day^−1^), DMSO (0.1%, diluted with PBS), and Rotenone (1.5 mg kg day^−1^) for five consecutive days. Next, the clinical severity of uveitis was confirmed using a slit lamp according to the five independent criteria and Caspi's criteria.^[^ ^80^ ^]^ The mice were then sacrificed, and the eyeballs were collected for subsequent experiments.

### Immunofluorescence Staining

The eyeballs were enucleated, and the retinas were cut into four‐leaf clover shapes. After permeabilization with 3% Triton X‐100 for 30 min and blocking with 5% goat serum for an hour, the flat‐mounts were incubated with IBA1 (1:500; Oasis, Zhejiang, China), (YY1) K183‐La (1:500; Cell Signaling, Massachusetts, USA), IRF1 (1:50; HUABIO, Hangzhou, China), IDO1 (1:500, Proteintech, Wuhan, China), SEMA4D (1:50; HUABIO), CCL5 (1:50; HUABIO), and STAT3 (1:50; HUABIO) iNOS (1:50; HUABIO), COX‐2 (1:50; HUABIO) and TNF‐α (1:50; Proteintech) antibodies at 4 °C overnight and washed in PBS three times. The retinas were incubated with Alexa‐594 (red) conjugated goat anti‐guinea pig IgG (1:1000; Oasis) and Alexa Fluor 488‐labeled Goat Anti‐Rabbit IgG (H+L) (1:500; Beyotime Institute of Biotechnology, Jiangsu, China) for 1 h. After washing with PBS, the retinas were added to the anti‐fade mounting medium, and images were captured using a confocal fluorescence microscope (Leica, Wetzlar, Germany).

### Hematoxylin and Eosin (H&E) Staining

Enucleated eyes were fixed and immersed in 4% glutaraldehyde (BioSharp, Hefei, China). After embedding the eyeballs in paraffin wax, eye sections were cut at a thickness of 4 µm, deparaffinized, and stained with hematoxylin and eosin (H&E).

### Cell Cultures and Cell Treatments

HMC3 cells obtained from the American Type Culture Collection (ATCC) were maintained in EMEM supplemented with 10% fetal bovine serum (FBS), 1% penicillin/streptomycin (all from Gibco; Thermo Fisher Scientific, Inc., USA) at 37 °C with 5% CO_2_. Thereafter, cells were seeded in 6‐well plates at 3 × 10^5^ cells well^−1^ and divided into two groups: the control group with no stimulation and the experimental group with LPS (1 µg mL^−1^) and IFN‐γ (500 ng mL^−1^). The cells were collected after 24 h. In the same way, HMC3 cells (stimulated using LPS+IFN‐γ) were randomly assigned to three groups, DMSO, DCA, and Rotenone were added separately for 24 h, and cells were harvested following the incubation period. DCA was added at a concentration of 20 mM and Rotenone was added at a concentration of 50 nM, according to previous studies.^[^ ^30^ ^]^

### Isolation of Primary Microglia

Postnatal (1–7 days) C57BL/6J mice were sacrificed. Brains were harvested, cut into pieces, and digested using trypsin for 30 min at 37 °C. Cells were then filtered through 70 µm filters, resuspended in F12/DMEM medium (containing 20% FBS, 1% penicillin‐streptomycin, and 10 ng mL^−1^ recombinant macrophage colony‐stimulating factor) and cultured in T75 flasks. 14 days later, cells were treated with 0.0625% trypsin for 30 min and 0.25% trypsin for another 10 min at 37 °C. The quality of the isolates was determined by staining for IBA1 and TMEM119 (microglia‐specific markers).

### Lentivirus Infection and A‐485 Treatment

Lentiviruses subcloned with cDNA of FLAG‐tagged YY1 WT or YY1 K183R mutant were constructed and purchased from Shanghai Genechem Co., Ltd. HMC3 cells were seeded in 6‐well‐plates at 1×10^5^ cells well^−1^ overnight and used for lentiviral infection. Then lentiviruses were added at a MOI of 30 for 24 h. Thereafter, the medium was replaced, and the cells were used for further experiments. In vitro, HMC3 cells cultured in 6‐well‐plates at 1×10^5^ cells well^−1^ were stimulated with LPS (1 µg mL^−1^), IFN‐γ (500 ng mL^−1^), A‐485(20 µM) for 24 hours as experimental group. In vivo, left eyes of EAU mice were intravitreally injected with A‐485 (200 µM, 1 µl eye^−1^) on day 9, right eyes were used as controls.

### Western Blotting (WB)

HMC3 cells were washed and lysed with RIPA buffer containing a protease inhibitor (Beyotime), and protein concentrations were determined using a BCA Protein Assay Kit (Beyotime). Equal amounts of 25 µg protein were loaded per lane into 8–12% SDS‐PAGE gels and transferred onto polyvinylidene fluoride (PVDF) membranes (Millipore, MA, USA) using the TransBlot Turbo system from Bio‐Rad. Membranes were blocked with Tris‐buffered saline with 0.1% Tween 20 (TBS‐T) containing 5% skim milk for an hour and then incubated with primary antibodies overnight at 4 °C. Primary antibodies included Pan‐Kla (1:1000, Proteintech); YY1 (1:1000, Proteintech); K183‐La (1:1000, Cell Signaling); INOS (1:1000, Proteintech); COX‐2 (1:1000, Proteintech); TNF‐α (1:1000, Wanleibio); p300 (1:500, Santa Cruz); HDAC6 (1:500, Santa Cruz); SIRT1 (1:500, Abcam); TIP60 (1:500, Santa Cruz); IRF1 (1:250, HUABIO), IDO1 (1:500, Proteintech), SEMA4D (1:250, HUABIO), CCL5 (1:250, HUABIO), STAT3 (1:250, HUABIO), MCP1 (1:500, Wanleibio), IL‐1β (1:500, Wanleibio), IL‐6 (1:500, Wanleibio). To confirm equalization of protein loading, membranes were probed with β‐actin antibody (1:1500, Servicebio). Next, the membranes were washed three times in TBST and incubated with horseradish peroxidase‐conjugated goat anti‐mouse IgG or HRP‐affinipure goat anti‐rabbit IgG secondary antibodies (1:10 000, Proteintech) for an hour at room temperature. Finally, the membranes were washed with TBST and visualized using an ECL kit (MedChemExpres, USA). Densitometry of the bands was performed using ImageJ software.

### Lactate Production

The lactate content in retinal tissues and microglial lysates was measured by the LA Content Assay Kit (Solarbio, BC2235). Using extracting solutions A and B, lactate was extracted from the same amounts of samples from different groups. Following the manufacturer's instructions, extracted lactate was added with color development solution and transferred into 96‐well plates. Lactate content was determined by measuring the absorbance at 570 nm. Each experiment was repeated thrice.

### Transwell Assay

Transwell chambers: 24‐well, 8.0‐µm pore membranes (Corning, NY, USA) were used according to the manufacturer's protocol. A total of 8.0 × 10^3^–1 × 10^4^ cells suspended in FBS‐free medium were seeded in the upper chamber, and 500 µl of complete medium was added to the lower compartment. After incubation for 24 h, LPS (1 µg mL^−1^, Sigma), IFN‐γ (500 ng mL^−1^, PeproTech), DCA (20 mM, Aladdin), DMSO (0.1%, Sigma), Rotenone (50 nM, Aladdin) were added to the lower chamber. One day later, cells were washed with PBS three times, fixed with 4% paraformaldehyde for 30 min, and stained with 0.25% crystal violet (Beyotime Institute of Biotechnology) for an hour. After removing the cells on the upper surface, the remaining cells on the lower surface were imaged and counted.

### EdU Assay

Cells were cultured in a 48‐well plate at 2×10^4^ cells per well and treated with LPS (1 µg mL^−1^, Sigma), IFN‐γ (500 ng mL^−1^, PeproTech), DCA (20 mM, Aladdin), DMSO (0.1%, Sigma) or Rotenone (50 nM, Aladdin), respectively. One day later, cells were treated with 500 µl of medium containing 100 µM EdU. After incubation at 37 °C, with 5% CO_2_ for 2 h, the cells were fixed with 4% paraformaldehyde for 10 min, washed three times with PBS, and incubated with 3% Triton‐X‐100 in PBS for 10 min. The cells were stained according to the manufacturer's instructions. Images of nine randomly selected areas in each group were captured using a fluorescence microscope (Leica, Wetzlar, Germany).

### Total RNA Extraction and Real‐Time Quantitative PCR

Total RNA was extracted from HMC3 cells (1 × 10^6^ cells) using Total RNA Extraction Reagent (Accurate Biology, Hunan, China). The manufacturers provided the required protocols. The isolated RNAs were reverse‐transcribed using the RT Master Mix for qPCR (gDNA digester plus) (Accurate Biology, Hunan, China). The cDNA samples were subjected to real‐time quantitative polymerase chain reaction (RT‐qPCR) using SYBR Green qPCR Master Mix (Accurate Biology, Hunan, China) and detected using an Applied Biosystems 7500 Real‐Time PCR System (Thermo Fisher Scientific). The selected genes were normalized to *GAPDH*. The PCR primers used are listed in **Table** **1**.

**Table 1:** Primers for RT‐qPCR.

Gene	DNA Sequence (5′→3′)
*STAT3*	Forward: CACCAAGCGAGGACTGAGCATC
	Reverse: AGCCAGACCCAGAAGGAGAAGC
*CCL5*	Forward: CAGCAGTCGTCCACAGGTCAAG
	Reverse: TTTCTTCTCTGGGTTGGCACACAC
*IDO1*	Forward: CATCTCACAGACCACAAGTCACAGC
	Reverse: CTTGGCAAGACCTTACGGACATCTC
*SEMA4D*	Forward: ACGGAGGTGTCTGTGGAGTATGAG
	Reverse: TCGGGCTTTCAGGAAGGAGGTC
*IRF1*	Forward: GCTACACAGTTCCAGGCTACATGC
	Reverse: TGCCACTCCGACTGCTCCAAG
*GAPDH*	Forward: CAGGAGGCATTGCTGATGAT
	Reverse: GAAGGCTGGGGCTCATTT

### CUT & Tag Analysis

In accordance with the manufacturer's instructions, the CUT&Tag assay was performed using a Hyperactive Universal CUT&Tag Assay Kit from Illumina (Vazyme, TD903‐01). Briefly, HMC3 cells were randomly distributed into two groups: the control group cultured in serum‐free medium, and the experimental group pretreated with LPS+IFN‐γ for 24 h. Collected cells were bound to Concanavalin A‐coated magnetic beads, permeabilized using Digitonin, and incubated with YY1 antibodies (CST, 46 395). Next, pA‐Tn5 transposase was added. DNA was extracted, amplified, and purified using transposon and tagmentation to construct a library. An Illumina NovaSeq 150PE platform was used for data analysis.

### Co‐immunoprecipitation (Co‑IP)

Immunoprecipitation of YY1 and p300 was performed using an immunoprecipitation kit (Abcam, ab206996). HMC3 cells were treated with LPS+IFN‐γ for 24 h. According to the manufacturer's instructions, cell lysates were collected and incubated with antibodies for 12 h at 4 °C. Forty microliters of protein A/G beads were pre‐washed and then incubated with beads for a further 2 h. After extensive washing, YY1 and p300 were detected by Western blotting. Antibodies used in Co‐IP included YY1 (1:1000, Cell Signaling), p300 (1:500, Santa Cruz), and a special secondary antibody, Veri‐Blot for IP Detection Reagent (HRP) (1:2000, Abcam) was used for IB after IP.

### Statistical Analysis

All data were shown as means ± SD. Statistical analyses were performed using SPSS 20.0 software (IBM, Chicago, IL, USA). The student's *t*‐test was used to compare the differences between the two groups. The mean ± SEM values of multiple groups were analyzed using a one‐way analysis of variance (ANOVA). The experiments were repeated independently using three different batches of cells or mice. Statistical analyses were performed using the Prism version 8.0 software (GraphPad, San Diego, CA, USA). * Denotes *p* < 0.05, ** denotes *p* < 0.01 and *** denotes *p* < 0.001.

## Conflict of Interest

The authors declare no conflict of interest.

## Author Contributions

J.H., X.W., and N.L. contributed equally to this work. J.H., X.W., N.L. and S.H. conceived and designed the experiments. J.H. X.W. and N.L. performed the experiments, analyzed the data and wrote the manuscript. W.F. helped to conduct in vivo experiments and contributed to data analysis. X.L., Q.Z. and J.L., H.Y. and M.T. helped to analyze CUT&Tag data. W.L. and Z.Z. helped to perform EdU assays. S.Z., X.L., H.Y. and M.T. helped to conduct immunofluorescence experiments. H.Y. and M.T. revised the manuscript. P.Y. supervised the experiments and revised the manuscript. S.H. conceptualized the study, supervised the experiments, acquired funding, and revised the manuscript.

## Supporting information

Supporting Information

Supporting Information

## Data Availability

The data that support the findings of this study are available from the corresponding author upon reasonable request.
