# Supplementary material for: YY1 Lactylation Aggravates Autoimmune Uveitis by Enhancing Microglial Functions via Inflammatory Genes
Source: Adv Sci (Weinh). 2024 Mar 17;11(19):2308031. doi: 10.1002/advs.202308031 (PMC11109619; doi:10.1002/advs.202308031)
Supplement: Supplementary file 1 — Supporting Information [file ADVS-11-2308031-s001.pdf]

ADVANCED SCIENCE
Open Access

Supporting Information

for *Adv. Sci.*, DOI 10.1002/advs.202308031

YY1 Lactylation Aggravates Autoimmune Uveitis by Enhancing Microglial Functions via Inflammatory Genes

Jiaxing Huang, Xiaotang Wang, Na Li, Wei Fan, Xingran Li, Qian Zhou, Jiangyi Liu, Wanqian Li, Zhi Zhang, Xiaoyan Liu, Shuhao Zeng, Hui Yang, Meng Tian, Peizeng Yang and Shengping Hou**

Figure.S1

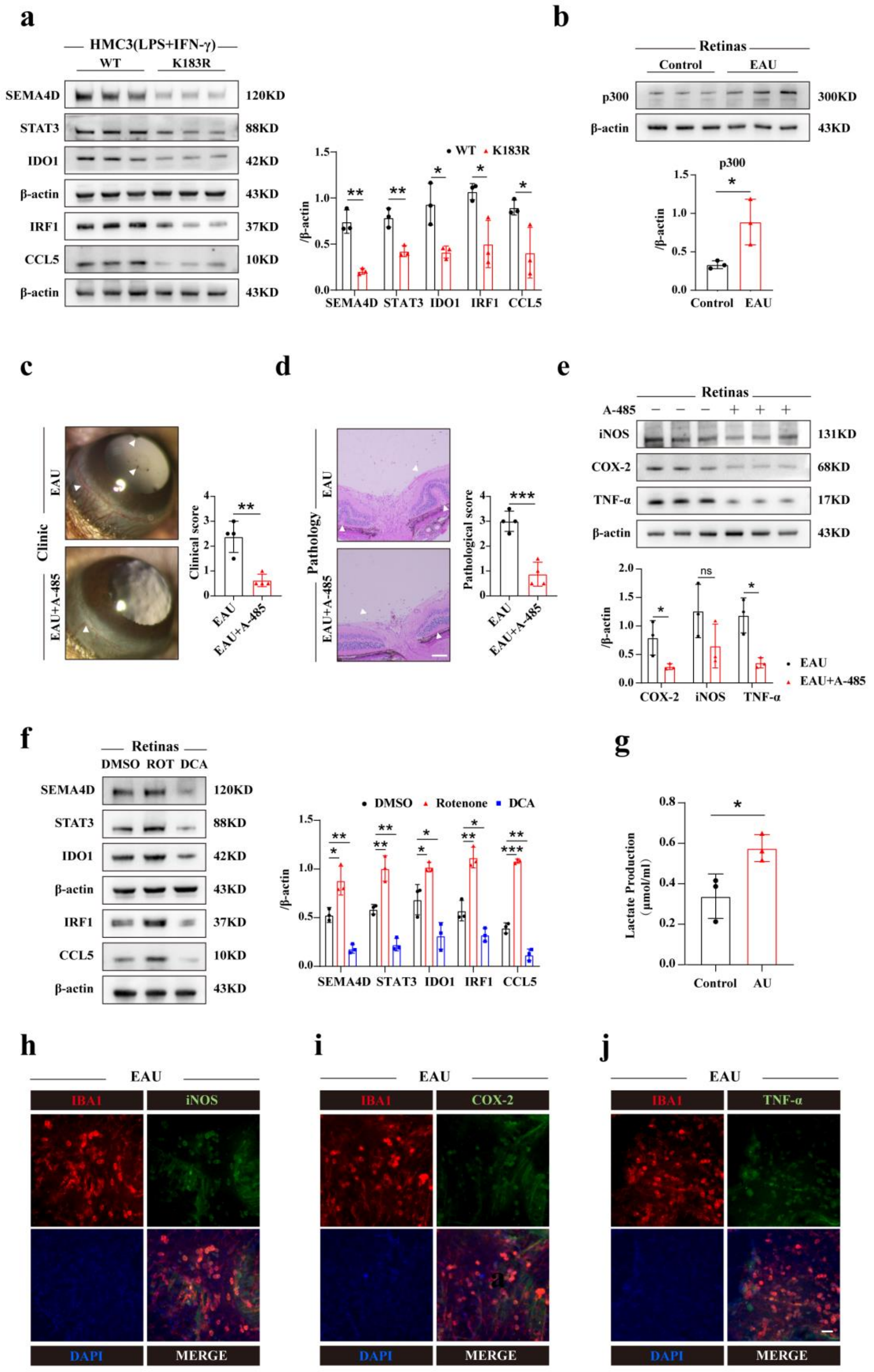

a
HMC3(LPS+IFN-γ)
WT
K183R
SEMA4D
STAT3
IDO1
β-actin
IRF1
CCL5
120KD
88KD
42KD
43KD
37KD
10KD
WT
K183R
/β-actin
b
Retinas
Control
EAU
p300
300KD
43KD
c
Clinic
EAU
EAU+A-485
Clinical score
d
Pathology
Pathological score
e
A-485
iNOS
COX-2
TNF-α
131KD
68KD
17KD
ns
f
DMSO
ROT
DCA
Rotenone
g
Lactate Production (μmol/ml)
AU
h
IBA1
DAPI
MERGE
i
j

Figure.S2

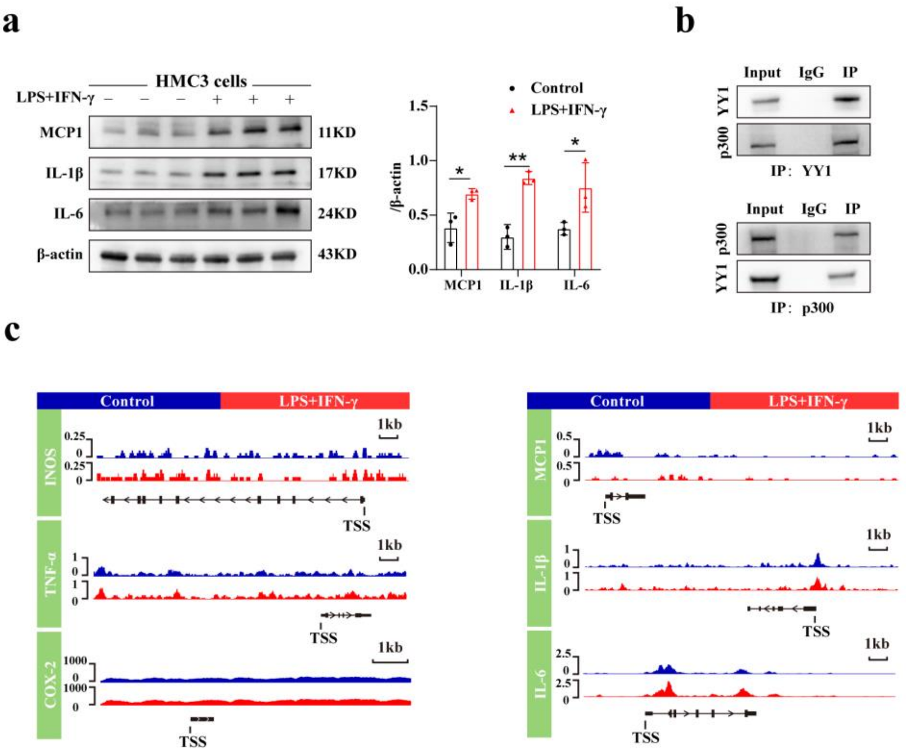
a
b
c
HMC3 cells
LPS+IFN-γ
MCP1
IL-1β
IL-6
β-actin
11KD
17KD
24KD
43KD
Control
LPS+IFN-γ
/β-actin
Input
IgG
IP
YY1
p300
IP: YY1
IP: p300
INOS
TNF-α
COX-2
TSS
1kb
